# Sialyltransferase and Neuraminidase Levels/Ratios and Sialic Acid Levels in Peripheral Blood B Cells Correlate with Measures of Disease Activity in Patients with Systemic Lupus Erythematosus and Rheumatoid Arthritis: A Pilot Study

**DOI:** 10.1371/journal.pone.0151669

**Published:** 2016-03-16

**Authors:** Lieh-bang Liou, Che-ching Huang

**Affiliations:** 1 Division of Rheumatology, Allergy, and Immunology, Chang Gung Memorial Hospital at Lin-kou, Kwei-san District, Tao-yuan City, Taiwan; 2 Chang Gung University College of Medicine, Kwei-san District, Tao-yuan City, Taiwan; Instituto Nacional de Ciencias Medicas y Nutricion Salvador Zubiran, MEXICO

## Abstract

**Objective:**

We attempted to determine whether the level of enzymes sialyltransferase (ST) and neuraminidase (Neu) and sialic acid (SIA) in patients with systemic lupus erythematosus (SLE) correlates with the SLE Disease Activity Index (SLEDAI) and in patients with rheumatoid arthritis (RA) correlates with the Disease Activity Score28 (DAS28).

**Methods:**

We examined cell-surface levels of ST6Gal-1, Neu1, ST3Gal-1, Neu3, α-2,6-SIA, and α-2,3-SIA by using fluorescent anti-enzyme antibodies, fluorescent-conjugated *Sambucus nigra* lectin, and fluorescent-conjugated *Maackia amurensis* lectin on blood cells in SLE and RA patients and assessed correlations of these levels with SLEDAI and with DAS28. Areas under the curve (AUC) were calculated for different variables against SLEDAI.

**Results:**

The B-cell ST3Gal-1/Neu3 ratio positively correlated with SLEDAI scores (ρ = 0.409 and *P =* 0.002, statistically significant after Bonferroni’ correction for multiple analyses.). It was supported by the inverse correlation of B-cell Neu3 levels with SLEDAI scores (ρ = −0.264, *P* = 0.048). The B-cell ST3Gal-1/Neu3 ratio against SLEDAI yielded an AUC of 0.689, which was comparable to that of anti-dsDNA levels at 0.635. In contrast, both ST3Gal-1 and Neu3 levels of RA B cells (r = 0.376, *P* = 0.013; r = 0.425, *P* = 0.005, respectively) correlated positively with high disease-activity DAS28 scores.

**Conclusion:**

B-cell ST3Gal-1/Neu3 ratios in SLE and B-cell ST3Gal-1 and Neu3 levels in RA with high disease-activity DAS28 scores correlated with disease activity measures and may be useful in monitoring disease activities.

## Introduction

Systemic lupus erythematosus (SLE) is an autoimmune disease characterized by polyclonal B-cell activation and the presence of many autoantibodies against a variety of autoantigens. The anti-dsDNA antibody is important in SLE and has long been used as a classification criterion and marker of disease activity, especially in lupus nephritis, though a study of clinical data [1] challenged the prognostic value of the anti-dsDNA antibody for disease flares. Most recently, the latter view is supported by an article that suggests that anti-dsDNA status does not seem to influence lupus disease activity [2]. Hence, elevated anti-dsDNA antibody with low complement levels have been used to monitor lupus activity for a long period of time, though their use is mainly restricted to predict outcome of lupus nephritis [3]. Nevertheless, a lot of articles on cytokines, chemokines, cell surface molecules, particular B-cell subsets, autoantibodies, and genetic (microRNAs) expression markers have been published to relate to lupus disease activity in the past 10 years [4–6]. The message is quite obvious: elevated anti-dsDNA antibody and/or low complement levels are not satisfactory enough for monitoring SLE disease activity in general and also for its diverse complications. In particular, lupus pathogenesis might also be affected by characteristics of B cells, by closely related immune cells (such as T cells and monocytes), or even by inflammatory cells such as polymorphonuclear (PMN) cells [7, 8]. Hence, immune cell abnormalities need also to be paid attention to.

A 1989 report showed that the increase in IgG binding of guinea pig peritoneal macrophages after neuraminidase treatment (which eliminates cell-surface sialic acid [SIA]) was due to increased affinity and not the number of Fcγ receptors [9]. Later, it was found that sialylated N-glycans on the cell surface suppressed the induction of phagocytosis, and that decreased expression of sialylation results in acquisition of the phagocytic ability in mouse monocytic cells [10]. Moreover, tolerogenic, immature dendritic cells had a higher α-2,6-SIA level, which was drastically downregulated by pro-inflammatory cytoines once the dendritic cells matured [11]. These results imply that phagocytes or antigen-presenting cells with low cell-surface SIA levels are more immunologically mature (more IgG binding or improved phagocytosis). Nevertheless, no such study has ever been done on cells in autoimmune diseases, such as in SLE and rheumatoid arthritis (RA).

The sialyltransferases (ST) are a family of enzymes that transfer sialic acid from cytidine 5’-monophosphosphate-sialic acid to a nascent glycoprotein or glycolipids [12, 13]. ST6Gal-1 mediates the transfer of sialic acid residue with an α-2,6-linkage to a terminal galactose residue of type 2 (Gal α1-4GlcNAc) disaccharide. ST6Gal-1 is likely localized in the Golgi apparatus, extracellular region, and cell membrane. The ST6Gal-1 gene is expressed in almost all human tissues [13]. It was implicated that different sialyltransferase levels can be used as a biomarker to discriminate normal and cancer patients [14, 15]. Moreover, ST6Gal-1 modifies sialylation of IgG Fc through the α1–3 Mannose branch-specific manner [16]. In particular, sialoadhesin-ligand (that is, SIA) levels on effector T cells correlated strongly with the degree of proteinuria in the NZB/W F1 murine model of spontaneous lupus [17]. The result suggested that this subset of SIA+CD4+ T cells was involved in inflammation and different from monocytes-macrophages and dendritic cells [9–11]. Nevertheless, no study has ever examined ST6Gal-1 on the peripheral blood cells of SLE patients or the associations of cell ST6Gal-1 levels with clinical and laboratory variables in SLE patients. Hence, we decided to investigate ST6Gal-1 and α-2,3-sialyltransferase (ST3Gal-1 catalyzes α-2,3 sialylation, which is also involved in the tumorigenesis and in T-cell apoptosis [18–20]), to determine if they are good markers of disease activity in SLE.

On the contrary, neuraminidases desialylate sialic acid in cells. Neuraminidase-1 (Neu1) may be involved in cellular signaling during the immune response. For example, Neu1 is required in early IL-4 production in T cells during contact with antigen-presenting cells [21], in the production of IgG1 and IgE by B cells [22], and in the regulation of phagocytosis in macrophages [23]. Neu1 is present in the lysosomal compartment and on the cell surface, whereas neuraminidase-3 (Neu3) is present only on the cell surface [24]. The ganglioside-specific sialidase, Neu3, may be important in cell-surface events, via modulation of gangliosides [24]. Similarly, no study has been done to explore possible relationship between Neu1 and Neu3 on lupus blood cells and lupus disease activity.

In summary, the ST6Gal-1/Neu1 pair works along the α-2,6-SIA pathway, and the ST3Gal-1/Neu3 pair works along the α-2,3-SIA pathway. An implication from above literature review was that adaptive immune cell’s sialyltransferases sialylate cell’s SIA to promote inflammation and sialylate secreted IgG to protect the host from inflammation. Neuraminidases have opposite effects as those of sialyltransferases on B cells and secreted IgG. Interestingly, chronic antigen exposure induced a 3-fold higher α-2,6-SIA/IgG Fc ratio, in contrast to acute antigen exposure [25]. Hence, a tentative explanation may be given as that the immune cell’s ST6Gal-1 level could increase along with a higher disease activity by chronic antigen exposure (eventually proceeds with increased cell and IgG sialylation) in human autoimmune diseases. While at the same time, the disease activity is still concomitantly high. Afterwards, increased IgG sialylation might down regulate antibody production of B cells through its binding to B-cell’s CD 22 molecules. Whether this immune mechanism might also play a feedback loop in autoimmune diseases remains unknown. Considering that lupus patients quite often have long-term autoantigen exposure, then, whether cell-surface sialyltransferase and neuraminidase expressions of peripheral blood cells correlate with clinical and laboratory variables in SLE patients is an intriguing topic and have not been determined yet. Similarly, those questions in patients with RA are also not clear and were studied together as a disease control for systemic lupus. We therefore examined the correlations of sialyltransferase and neuraminidase expressions on blood cells with clinical and laboratory disease parameters in lupus and RA patients.

## Patients and Methods

### Ethics statement

The study was approved by Chang Gung Institutional Review Board (Tao-yuan, Taiwan) and had therefore been performed in accordance with the ethical standards laid down in an appropriate version of the 1964 Declaration of Helsini.

### Patient enrollment and recording of clinical and laboratory data

115 lupus patients who fulfilled the 1997 American College of Rheumatology criteria for SLE and gave written informed consent for participation in this study were randomly selected after they agreed to blood collection for the present analysis. 157 RA patients who fulfilled the 1987 American College of Rheumatology criteria for RA were similarly enrolled. Twenty-five milliliters of blood were collected from each patient at baseline and also after 1, 3, and 6 months from a small number of patients. The SLE Disease Activity Index (SLEDAI) was calculated by using SLEDAI-2K [26] and data on the different organ systems involved and serum C3, C4, and anti-dsDNA antibody levels were obtained for each patient. In order to capture more occurrence of different lupus manifestations in lupus patients, we tried to collect as many events as possible to detect possible differences of enzyme levels/ratios between those having separate lupus manifestations and those not at different time points as mentioned above. Similarly, the Disease Activity Score28 (DAS28) was recorded for RA patients. 31 healthy controls were also included, after our Hospital personnel gave informed consent, for verifying our observation in disease groups being specific.

### Cell separation and plasma/serum collection

Blood was collected in tubes containing ethylenediaminetetraacetic acid (EDTA), and plasma and cells were centrifuged and separated. These cells were then separated by Ficoll-Hypaque density-gradient centrifugation and processed as previously described [27] to obtain peripheral blood mononuclear cells (PBMCs). PMN cells were obtained from the bottom of the Ficoll-Hypaque layer [28].

### Detection of cell-surface sialic acid, ST6Gal-1, ST3Gal-1, Neu1, and Neu3 on monocytes, B and T lymphocytes, and PMN cells

PBMCs in 100 μl phosphate-buffered saline (PBS) with 1% bovine serum albumin (BSA) (Sigma, MO, USA) were stained with either phycoerythrin (PE) mouse anti-human CD19 (clone: HIB19) for B cells, PE mouse anti-human CD3 (clone: HIT3a) for T cells, or PE mouse anti-human CD14 (clone: M5E2) for monocytes (used as manufacturer recommendations, all from BD Pharmingen, Mountain View, CA, USA) and stored at room temperature under light protection for 1 hr. Their individual isotype control was PE mouse IgG1 *κ* isotype control (clone: MOPC-21) (for anti-CD19 and anti-CD14) or PE mouse IgG2a isotype control (clone: G155-178) (for anti-CD3) (used as manufacturer recommendations, both from BD Pharmingen, Mountain View, CA, USA). Fluorescein isothiocyanate (FITC)-conjugated *Sambucus nigra* lectin (SNA; specifically binds α-2,6-SIA) at 20μg/ml per test in 100 μl PBS with BSA or FITC-conjugated *Maackia amurensis* lectin (MAA; specifically binds α-2,3-SIA) at 40μg/ml per test (both from EY Laboratories, Inc., San Mateo, CA, USA) was added as previously described [11] with FITC-mouse IgG1 *κ* isotype control (used as manufacturer recommendations, BD Pharmingen). And the cells were stored at room temperature under light protection for 1 hr. In addition, mouse monoclonal IgM anti-ST6Gal-1 (anti-CD75, clone: B-L5) at 7.5μl out of a 2ml-vial (the manufacturer recommendation is 10μl), rabbit polyclonal IgG anti-ST3Gal-1 at 0.6μg/ml per test, rabbit polyclonal IgG anti-Neu1 at 51μg/ml per test, or rabbit polyclonal IgG anti-Neu3 at 0.4μg/ml per test (all from Abcam, Cambridge, MA, USA) was added. Mouse IgM isotype control (clone: MOPC1041) at 4μg/ml per test (Sigma, St. Louis, MO, USA) and rabbit polyclonal IgG (JacsonImmunoResearch, West Grove, PA, USA) at 2μg/ml per test were used as the controls.

After washing and centrifugation (Eppendorf centrifuge 5810R) at 644x *g* for 5min, cells in 100 μl PBS were then stained with either allophycocyanin (APC)-rat monoclonal antibody (1B4B1) to mouse IgM at 2μg/ml per test or APC-goat polyclonal antibody to rabbit IgG at 2μg/ml per test (all from Abcam, Cambridge, MA, USA) at room temperature under light protection for 1 hr. Using similar washing and centrifugation techniques, we resuspended cells in 500 μl PBS at 4°C for flow cytometric analysis by the BD FACSCalibur System. The minimal number of cells collected was 20,000. The FlowJo 7.6.1 program (FlowJo, LLC, Ashland, Oregon, USA) was used to analyze all flow cytomery data.

All examined parameters were: CD19, CD3, CD14, α-2,6-SIA, α-2,3-SIA, ST6Gal-1, ST3Gal-1, Neu1, and Neu3.

### Statistical analyses

The SPSS 16.0 software pacage was used for data analysis. We estimated correlations of cell-surface levels of ST6Gal-1, ST3Gal-1, Neu1, Neu3, α-2,6-SIA, and α-2,3-SIA (all independent variables) with clinical outcome variables (SLEDAI, which includes arthritis, sin rash, etc.; DAS28 scores) and laboratory outcome variables (C3, C4, and anti-dsDNA antibody levels and proteinuria; ESR, CRP) for SLE and RA. DAS28 scores were calculated based on the universally used equation (including ESR, TJC, SJC, and GH) as described [29]. Spearman correlation coefficients (ρ) were used when outcome variables (SLEDAI and DAS28) were not normally distributed, and Pearson correlation coefficients (r) were used when outcome variables were normally distributed. When no obvious independent-dependent relationship was found, Spearman correlation coefficients (or Mann-Whitney U test for comparisons) were calculated when any one of two variables was not normally distributed; Pearson correlation coefficients were used when both variables were normally distributed. A *P* value of < 0.05 was considered to indicate statistical significance. Moreover, a *P*-value between 0.05 and 0.10 was considered to indicate a trend [29]. Bonferroni’s correction was used for multiple analyses in particular sets of data.

Receiver-operating characteristic (ROC) curves for the B cell ST3Gal-1/Neu3 ratio and the anti-dsDNA level against SLEDAI were plotted to calculate areas under the curve (AUCs).

## Results

### Demographic characteristics of SLE and RA patients

In total, 115 consecutive patients with SLE [The mean age was 41.9±11.6 (range 21–69)] and 157 with RA [The mean age was 53.0±11.7 (20–80)] were enrolled (Table 1). The mean age of 31 healthy control persons was 30.1±9.1 (range 23–58). Detailed medicines that were taken by RA and SLE patients were given in S1 and S2 Tables.

**Table 1:** Demographic data of enrolled SLE and RA patients.

	SLE	RA	Controls
No. of subjects (F:M ratio)	115 (105:10)	157 (127:30)	31 (26:5)
Disease duration (months)	109.3±63.8 (1–336)	96.6±85.4 (2–417)	_
Number of arthritic joints	_	6.7±5.6 (0–26)#	_
Patients having proteinuria	19	_	_
Patients having arthritis	6	155	_
Patients having skin rash	3	**_**	_
Patients having vasculitis	1	**_**	_
C3c (mg/dl)	87.1±25.9 (18.6–188.0)	_	_
C4 (mg/dl)	17.7±12.3 (1.50–122.0)	_	_
Anti-dsDNA (mg/dl)	171.3±128.9 (35.3–546.9)	_	_
Rheumatoid factor (IU/ml)	_	137.8±246.2 (1.2–1200.0)	_
ESR (mm/hr)	_	24.6±20.9 (2.0–101.0)	_
CRP (mg/L)	_	9.3±17.2 (0.2–133.2)	_
SLEDAI	3.4±2.6 (0–12.0)	_	_
DAS28	_	4.3±1.3 (1.9–7.3)	_
Prednisolone and equivalent* (mg/day)	11.7±8.4 (2.5–50.0): 76.5%	6.0±3.1 (2.5–15): 34%	_
One DMARD*	24.4%	19.7%	_
Two or more DMARDs*	73.0%	72.0%	_
Biologicals*	-	24.8%	_

SLE: systemic lupus erythematosus; RA: rheumatoid arthritis; Controls: healthy controls; F:M ratio: female:male ratio; SLEDAI: SLE disease activity index; DAS28: disease activity score28; DMARD, disease-modifying anti-rheumatic drugs. #Two RA patients had no arthritic joints. Normal ranges: C3: 90–180; C4: 10–40; anti-dsDNA: <92.7; ESR: <15; CRP: <3.0. Data are expressed as mean±S.D. (ranges).

*percentages of patients have taken that particular medicine for more than 3 months (those durations not fulfilled were not counted).

### Correlation of sialyltransferases and neuraminidases with lupus activity

**Fig 1:**
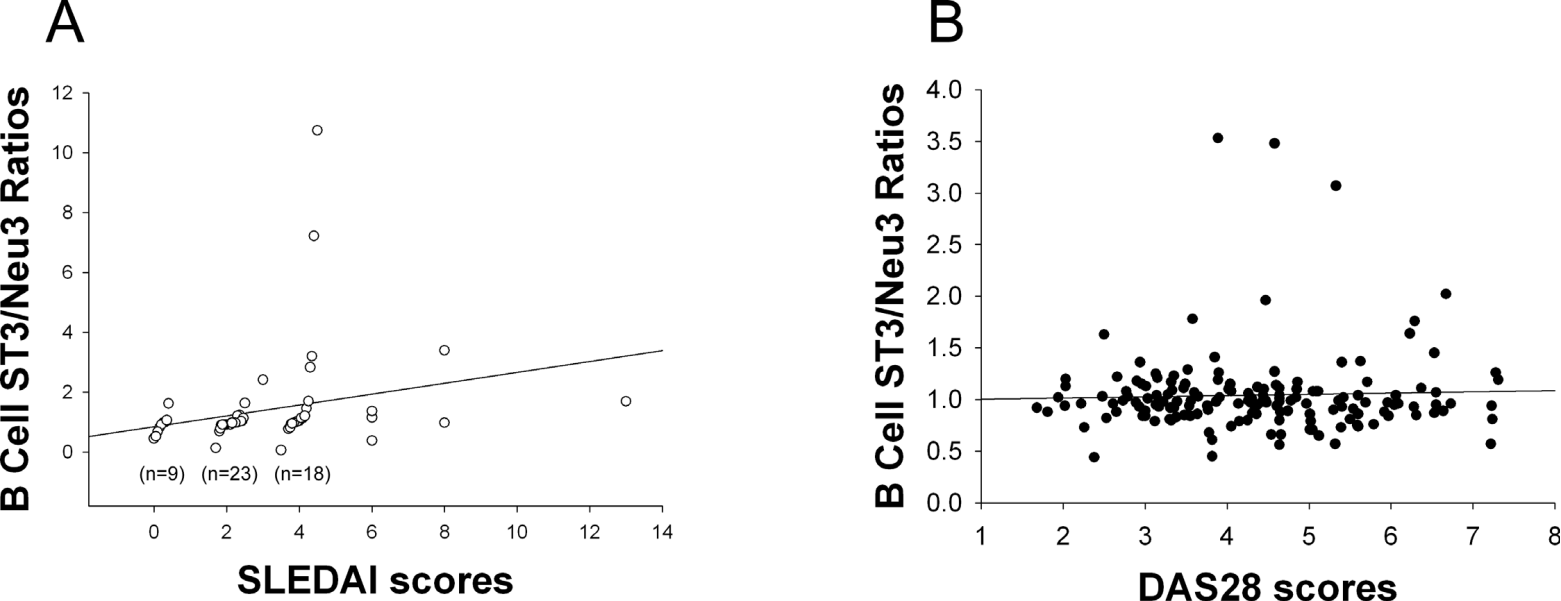
Correlation between the B-cell ST3Gal-1/Neu3 ratios and SLEDAI or and DAS28. B cells were stained as described in Patients and methods. The B-cell ST3Gal-1/Neu3 ratio indicated the ratio of the mean fluorescence intensity (MFI) of the B-cell’s ST3Gal-1 staining results divided by the MFI of the same B-cell’s Neu3 staining results. (A) Positive correlation between the B-cell ST3Gal-1/Neu3 ratio and SLEDAI. SLEDAI: systemic lupus erythematosus disease activity index; In order to have distinctive circles in the graph, those circles with SLEDAI scores equal to 0, 2, and 4 were adjusted on one-to-two figure(s) to the right of the decimal point either above or below 0, 2, and 4. The correlation was ρ = 0.409 and *P* = 0.002 (n = 57). It was statistically significant when compared with a significant *P*-value = 0.0125 after Bonferroni’s correction was done with no significant correlation was found for correlating the ST3Gal-1/Neu3 ratio of T and PMN cells, and monocytes with SLEDAI scores. (B) Correlation between B-cell ST3/Neu3 ratios and DAS28 (disease activity score28) at r = 0.039 and *P* = 0.631 (n = 157).

**Fig 2:**
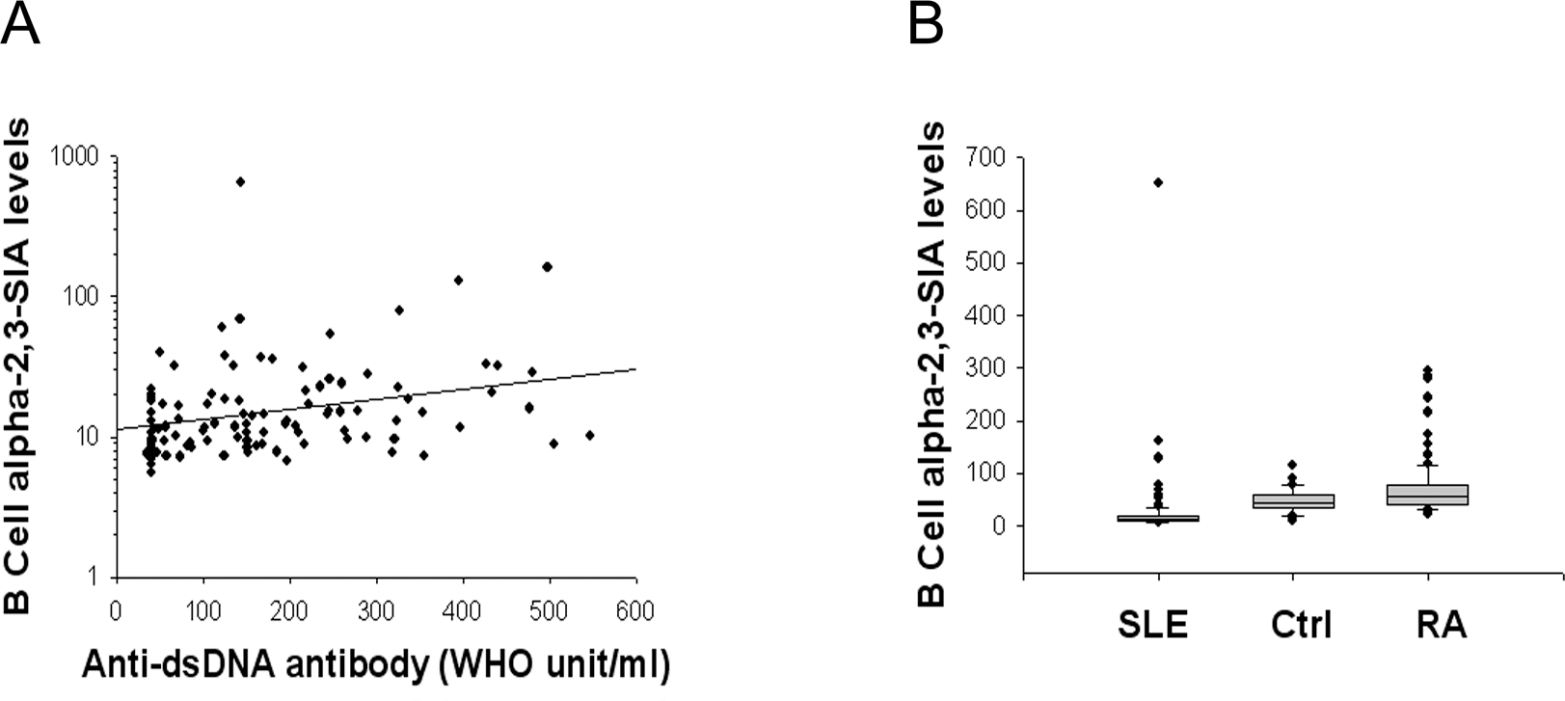
B-cell α-2,3-sialic acid (SIA) levels in systemic lupus (SLE), healthy controls (Ctrl), and rheumatoid arthritis (RA). B-cell α-2,3-SIA levels indicated the mean fluorescence intensity of the B-cell’s SIA staining results. (A) Positive correlation between B-cell α-2,3-SIA levels and serum anti-dsDNA antibody levels (WHO unit/ml) (n = 106). Using a staining method similar to that described in Fig 1, the second layer of staining used FITC-conjugated *Macckia amurensis* lectin to bind cell surface α-2,3-SIA. WHO: World Health Organization. The correlation was ρ = 0.313 and *P* = 0.001. (B) Comparison of B-cell α-2,3-SIA levels among various groups. SLE (n = 106) vs. Ctrl (n = 31) gave *P*<0.001; SLE vs. RA (n = 157) rendered *P*<0.001; RA vs. Ctrl gave *P* = 0.011. All were significant when Bonferroni’ correction was done to make a significant *P*-value at 0.0167.

**Fig 3:**
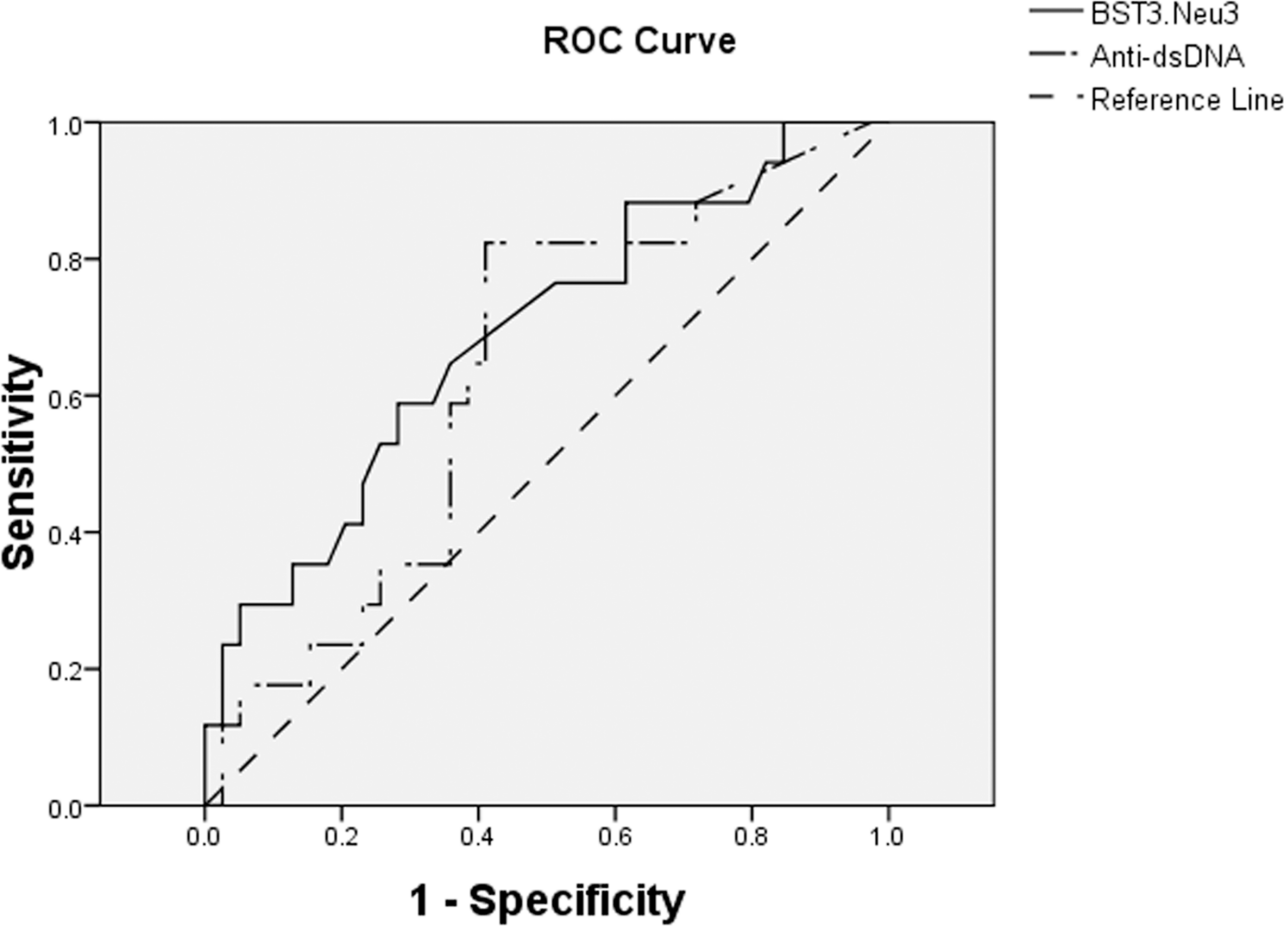
ROC curves of cell ST3Gal-1/Neu3 raios and serum anti-dsDNA levels against SLEDAI scores. The ROC curve of the B-cell ST3Gal-1/Neu3 ratio (Bst3neu3) against SLEDAI (n = 57) yielded an AUC of 0.689, which was comparable to the AUC of 0.635 for the anti-dsDNA level (Anti-dsDNA) against SLEDAI (n = 57).

The B-cell ST3Gal-1/Neu3 ratio was significantly positively correlated with SLEDAI scores (Fig 1A), similarly as was the former’s correlation with the serum anti-dsDNA antibody level (r = 0.531, *P*<0.001). This result was supported by the inverse correlation of B-cell Neu3 levels with SLEDAI scores (ρ = −0.264, *P* = 0.048) and the positive correlation of the B-cell α-2,3-SIA (which is produced by ST3Gal-1) with serum anti-dsDNA antibody levels (Fig 2A). Lupus B-cell α-2,3-SIA levels were lower than those of healthy controls and of patients with rheumatoid arthritis (Fig 2B). Additionally, the ROC curve of the B-cell ST3Gal-1/Neu3 ratio against SLEDAI yielded an AUC, which was comparable to the AUC of the anti-dsDNA level against SLEDAI (Fig 3). These results suggest that the B-cell ST3Gal-1/Neu3 ratio correlated with SLEDAI scores by using two different methods (linear correlation and ROC analyses) and could possibly be used as a surrogate marker of disease activity in patients with systemic lupus, pending further validation.

In contrast, we found no significant correlation of blood cell-surface ST6Gal-1 levels or ST6Gal-1/Neu1 ratios with SLEDAI, C3c, C4, or anti-dsDNA levels (r or ρ*<* 0.200 or > −0.200 for all; all original data are not shown). One of the exceptions was that the B-cell ST6Gal-1/Neu1 ratio was inversely correlated with the serum C4 level (ρ = −0.224, *P* = 0.036). Moreover, B-cell ST6Gal-1 (which produces -2,6-SIA) and B-cell α-2,6-SIA levels were inversely correlated with serum C3c levels (ρ = −0.207 and *P* = 0.049 and ρ = −0.279 and *P* = 0.007, respectively). Similarly, T-cell α-2,6-SIA levels were inversely correlated with serum C3c levels (ρ = −0.276, *P* = 0.008). B-cell and monocyte ST6Gal-1 levels were likewise inversely correlated with serum C4 level (ρ = −0.286 with *P* = 0.006 and ρ = −0.210 with *P* = 0.045, respectively).

Hence, ratios and levels of B-cell ST3Gal-1/Neu3 and ST6Gal-1/Neu1 ratios correlated moderately and modestly with measures of SLE activity, respectively.

### Correlation of sialyltransferases and neuraminidases between different cell types in lupus

The B-cell ST6Gal-1/Neu1 ratio significantly correlated with the ST6Gal-1/Neu1 ratio of T cells, monocytes, and PMN cells (r = 0.367, *P*<0.001; ρ = 0.470, *P*<0.001; and ρ = 0.406, *P*<0.001, respectively; all original data are not shown). The B-cell ST6Gal-1 level was significantly positively correlated with the ST6Gal-1 level in T cells, monocytes, and PMN cells (ρ = 0.760, *P*<0.001; ρ = 0.534, *P*<0.001; and ρ = 0.455, *P*<0.001, respectively). A similar trend was observed in the correlation of the B-cell Neu1 level with the Neu1 level of T cells, monocytes, and PMN cells (ρ = 0.421, *P*<0.001; ρ = 0.490, *P* = 0.001; and ρ = 0.282, *P* = 0.007, respectively).

Moreover, the T-cell ST6Gal-1 level correlated with the ST6Gal-1 level in monocytes and PMN cells (r = 0.574, *P*<0.001, and ρ = 0.426, *P*<0.001; respectively; all original data are not shown). Similarly, the monocyte ST6Gal-1 level correlated with the ST6Gal-1 level in PMN cells (ρ = 0.325, *P* = 0.002). These statistical significance correlations were still valid, after Bonferroni’s corrections were done. Hence, ST6Gal-1 and Neu1 levels, and ST6Gal-1/Neu1 ratios were paralleled in various blood cells from individual lupus patients. Unexpectedly, no such correlation was found in ST3Gal-1 and Neu1 expression between B cells and other cells.

### Comparison of sialyltransferase and neuraminidase levels in lupus subgroups

**Fig 4:**
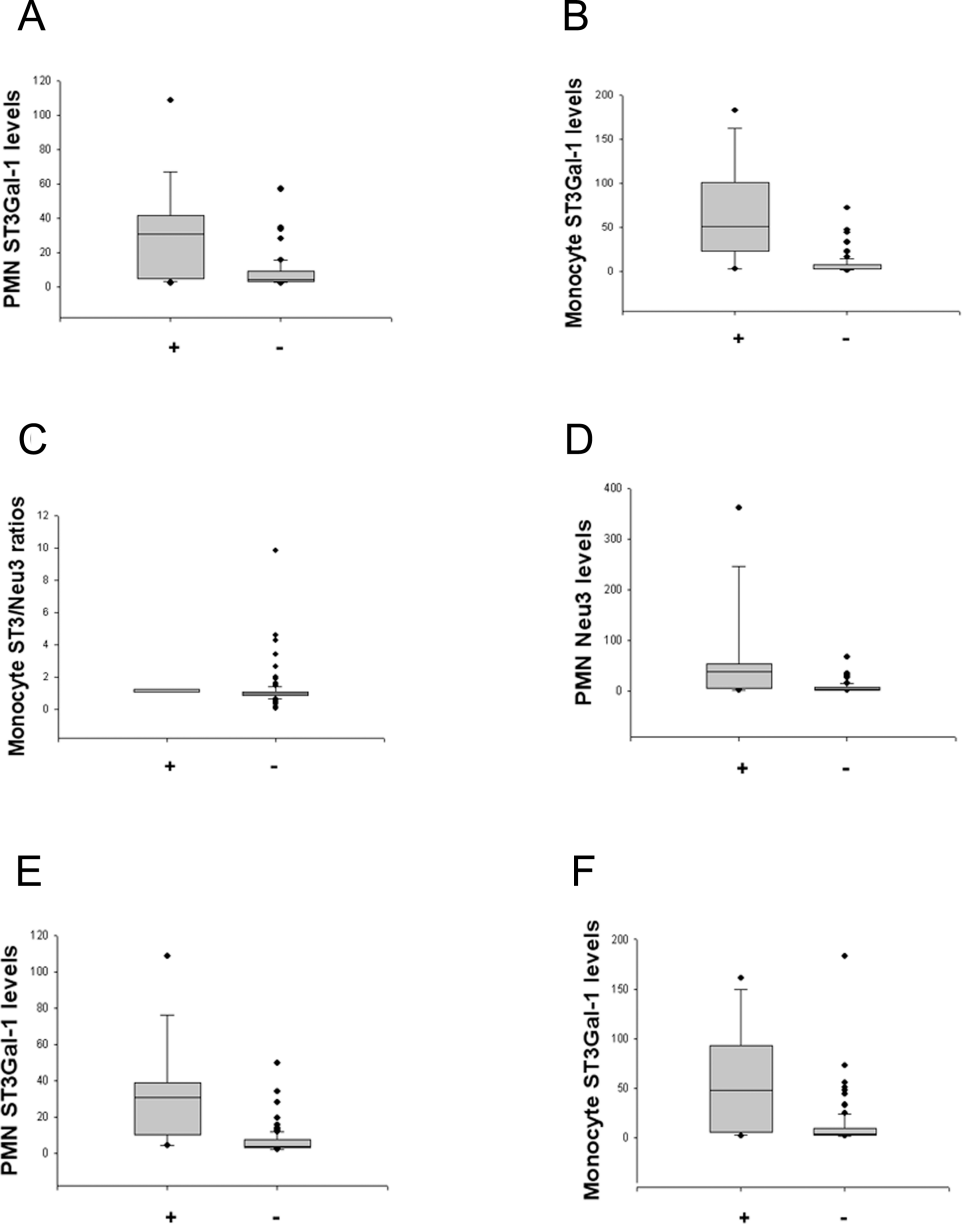
Different expression levels of sialyltransferases and neuraminidases in separate lupus manifestations and disparate disease activity categories. Polymorphonuclear cells (PMN) (or monocyte) ST3Gal-1 (or Neu3) levels indicated the mean fluorescence intensity (MFI) of PMN’s (or monocyte’s) ST3Gal-1 (or Neu3) staining results. Moreover, monocyte ST3Gal-1/Neu3 ratios indicated the ratio of the MFI of respective cell’s ST3Gal-1 staining results divided by the MFI of the same monocyte’s Neu3 staining results. (A) Higher PMN ST3Gal-1 levels were found in the proteinuria subgroup (+) (n = 18) vs. the non-proteinuria subgroup (-) (n = 61) (*P*<0.001). (B) Higher monocyte ST3Gal-1 levels were found in the proteinuria subgroup (+) (n = 18) vs. the non-proteinuria subgroup (-) (n = 62) (*P* <0.001). (C) Higher monocyte ST3Gal-1/Neu3 ratios were noted in the arthritis subgroup (+) (n = 7) vs. the no-arthritis subgroup (-) (n = 105) (*P* = 0.016). (D) Higher PMN Neu3 levels were found in the proteinuria subgroup (+) (n = 18) vs. the non-proteinuria subgroup (-) (n = 61) (*P*<0.001). (E) Higher PMN ST3Gal-1 levels were noted in the SLEDAI > 7 subgroup (+) (n = 16) vs. the SLEDAI ≤ 7 subgroup (-) (n = 91) (*P*<0.001). (F) Higher monocyte ST3Gal-1 levels were noted in the SLEDAI > 7 subgroup (+) (n = 17) vs. the SLEDAI ≤ 7 subgroup (-) (n = 96) (*P* <0.001).

In order to capture more occurrences of different lupus manifestations in lupus patients, we had collected as many events as possible at different time points and pooled them together for further analysis as described in Patients and methods. Pooling of individual-visit measurements yielded 210 individual data for comparison of event-measurements by category of organ involvement (i.e., more vs. fewer events with specific organ involvement) and category of disease activity (more vs. fewer events with greater disease activity). Both higher PMN ST3Gal-1 levels and higher monocyte ST3Gal-1 levels were found to exist in the proteinuria subgroup than those in the non-proteinuria subgroup (Fig 4A & 4B). Higher monocyte ST3Gal-1/Neu3 ratios were noted in the arthritis subgroup than those in the no-arthritis subgroup (Fig 4C). Higher PMN ST3Gal-1 levels and higher monocyte ST3Gal-1 levels were noted in the SLEDAI > 7 subgroup than those in the SLEDAI ≤ 7 subgroup (Fig 4E and 4F). So as were for higher PMN Neu3 levels and higher monocyte Neu3 levels in the proteinuria subgroup (Fig 4D and S2A Fig). Furthermore, higher PMN Neu3 levels and higher monocyte Neu3 levels were noted in the SLEDAI > 7 subgroup than those in the SLEDAI ≤ 7 subgroup (S2B and S2C Fig). These results indicate that ST3 and Neu3 levels or ratios in peripheral blood monocytes and PMN cells, may be used to differentiate between patients with and without two lupus symptoms (proteinuria and arthritis) and between greater and lesser disease activity (in SLEDAI scores).

### Correlation of sialyltransferases and neuraminidases with RA disease activity

**Fig 5:**
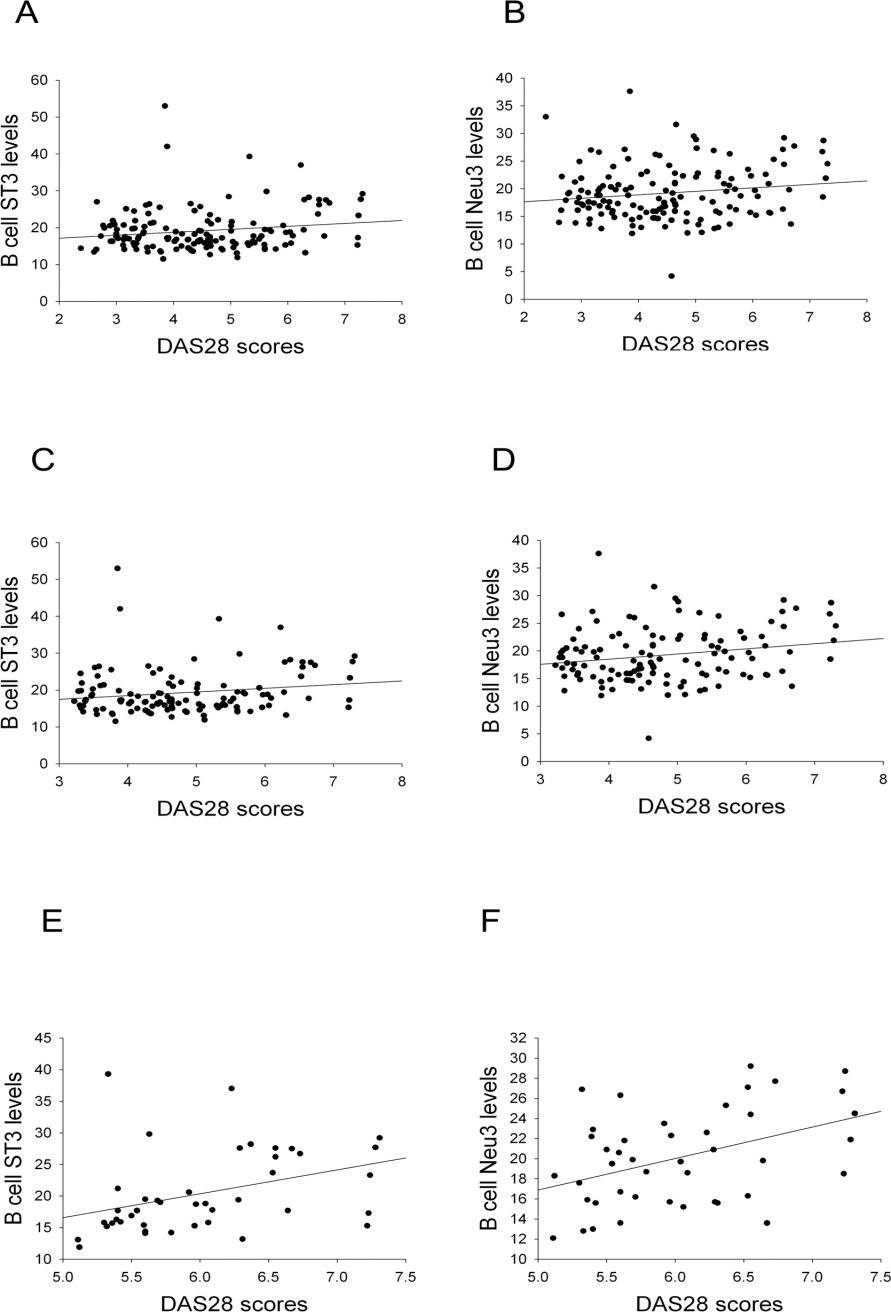
The association of B cell sialyltransferase and neuraminidase levels with DAS28 scores in RA patients. ST3: ST3Gal-1, sialyltransferase 3; Neu3: neuraminidase 3; DAS28: disease activity score 28; RA: rheumatoid arthritis. B cell ST3 and Neu3 levels indicated the mean fluorescence intensity of the B-cell’s ST3Gal-1 and Neu3 staining results. Correlation coefficients and *P*-values for: (A) r = 0.165, *P* = 0.047; (B) r = 0.155, *P* with a trend (n = 146 of RA patients in non-remission in A and B); (C) r = 0.170, *P* with a trend; (D) r = 0.201, *P* = 0.026 (n = 123 of RA patients with moderate and high disease activity in C and D); (E) r = 0.376, *P* = 0.013; (F) r = 0.425, *P* = 0.005 (n = 43 of RA patients with high disease activity in E and F).

In 157 RA patients, B-cell ST3/Neu3 ratios did not correlate with DAS28 scores (Fig 1B). Such correlation was further analyzed in RA patients with various categories of disease activity (remission and non-remission designate RA patients with DAS28 scores <2.6 and ≧2.6, respectively; moderate and high disease activity indicate RA patients with DAS28 scores >3.2 but ≤5.1, and >5.1, respectively). It was interesting to find that B cell ST3Gal-1 (Fig 5E) and Neu3 levels (Fig 5D and 5F) were significantly correlated with DAS28 scores in RA patients with moderate and high disease activity. Moreover, B-cell α-2,3-SIA levels (which is made by B-cell ST3Gal-1) correlated with DAS28 at t = 2.317 and *P* = 0.022 in multivariable analysis (by linear regression), which is analyzed together with α-2,3-SIA levels of T cells, monocytes, and PMNs against DAS28.

## Discussion

Kaneo *et al* reported that anti-platelet antibodies with high and low α-2,6-SIA levels varied in their capacity to cause thrombocytopenia [30]. Moreover, ST6Gal-1-deficient mice (together with α-2,6-SIA-deficient lymphocytes) exhibited reduced B cell proliferation and decreased antibody production in response to T-independent and T-dependent antigens [31]. These results indicate that low or no B lymphocyte ST6Gal-1 synthesis, together with low or no α-2,6-SIA production, is associated with a reduced immunoinflammatory effect. Whether a similar phenomenon exists in SLE was the subject of the present study.

This is the first study to report that changes in SLEDAI paralleled to ratios of ST3Gal-1/Neu3 enzymes in B cells (Fig 1A). These findings were supported by the results in Figs 2 and 3. Taken together, these findings suggest that the ST3Gal-1 and Neu3 enzymes in B cells are potential marers of lupus activity. A future large- population study is needed to confirm such viewpoint. Nevertheless, it is unclear whether the B cell ST3Gal-1 and Neu3 enzymes have an effect on α-2,3-SIA contents of secreted anti-dsDNA, despite it was found that α-2,3-SIA linage added on IgG Fc fragments do not confer IgG’s anti-inflammatory effect [32]. Future *in vitro* studies of lupus B cells are necessary in order to resolve this question.

The higher ST6Gal-1 level and ST6Gal-1/Neu1 ratio in B cells potentially suggest greater lupus activity, due to their compatibility with low complement levels, pending for a large-population study to support it. Nevertheless, the findings might obtain support from the report showing that chronic antigen exposure induced a 3-fold higher α-2,6-SIA/IgG Fc ratio, in contrast to acute antigen exposure [25]. However, they did not examine changes of the ST6Gal-1 enzyme on mouse B cells. Hence, a tentative explanation may be given as that the B-cell ST6Gal-1 level increases along with a higher disease activity by chronic antigen exposure (eventually proceeds with increased cell and IgG sialylation) in SLE and RA. While at the same time, the disease activity is still concomitantly high. Our findings on B cells, similar to those of chronic antigen exposure, but were obviously contrasted with those of acute antigen exposure, which induced depressed IgG sialylation [25]. In turn, secreted IgG’s increased SIA and B-cell's increased SIA possibly induce inhibition of other B-cell’s production of autoantibodies, via conjugation with CD22 on B cells, to form a cycle of reparation. The tentative explanation waits for future investigation to confirm or modify. Furthermore, it is unclear whether lupus B cell ST6Gal-1/Neu1 enzymes affect α-2,6-SIA contents of anti-dsDNA antibodies (a component of SLEDAI). It requires clarification in a future study, perhaps in an *in vitro* study of lupus B cells.

The strength of the present study was that we are the first to report that cell-surface sialyltransferase and neuraminidase levels in peripheral blood correlated with SLEDAI and DAS28 scores, hence, may be used as potential marers of lupus and RA disease activity. First, we demonstrated that changes in the ratios of B-cell ST3Gal-1 and Neu3 correlated with changes in SLEDAI. Second, B-cell ST3Gal-1/Neu3 ratios predict SLEDAI as strong as that of serum anti-dsDNA levels to predict SLEDAI (Fig 3). Third, B-cell ST6Gal-1 levels and ST6Gal-1/Neu1 ratios may indicate the presence of lupus activity pending further exploration, due to the correlations of these variables with low serum C3c and C4 levels. Fourth, the positive correlations of ST6Gal-1 and Neu1 levels on B, T, and PMN cells and monocytes in lupus, both overall and in individuals, is likely a new finding with unexplained reasons. Fifth, high ST3Gal-1 and Neu3 levels of PMN cells and monocytes were possible indicators of the presence of lupus proteinuria and a high SLEDAI score. In particular, the present study represents a collection of real-world patients, unlike a narrow and restricted range of patients in clinical trials. Last, this study offers a new research direction from bedside to bench.

This study had limitations. First, the apparent clinical usefulness of our present results regarding B cells and PMN cells requires confirmation in a much larger cohort population to determine their clinical usefulness across different time points, in addition to the present cross-sectional study (only a small number of patients were followed longitudinally in this study). Then, whether B cell ST3Gal-1/Neu3 and ST6Gal-1/Neu1 pairs can be used as a clinical marker of activity will be determined. Second, only when the B cell ST3Gal-1/Neu3 ratio is validated by radiographic examination and assessment of patients’ physical function will it be called a clinical marker of activity in RA patients, especially across different categories of DAS28 scores. Last, there is no mechanistic investigation yet to explain the different expression of ST3Gal-1 and Neu3 between SLE and RA patients. All these weakness needs to be re-evaluated in a future and large-cohort study.

At the human disease level, serum levels of interferon-controlled chemokines (e.g. CCL19 and CXCL10) have been found to correlate with current lupus activity, disease flare-up, and disease remission [33]. Though CCL19 and CXCL10 levels differentiated patients with active SLE (with SLEDAI ≥6) from inactive SLE (with SLEDAI ≤2), similar comparison results did not occur between patients with intermediate (with SLEDAI between > 2 and <6) and low lupus activity. In particular, this report did not demonstrate a linear correlation (or a high area-under-the curve in receiver-operating characteristic analysis) of chemokine levels or scores with (or against) SLEDAI scores, being a significant drawback for clinical practice.

Moreover, erythrocyte-C4d, an erythrocyte-bound complement activation product, has been shown to correlate well with lupus disease activity and suggested to use for monitoring lupus activity [34]. It was noted that the erythrocyte-C4d levels correlated with modified SELENA-SLEDAI scores in an analysis with a linear mixed-effects model. The perspective of using erythrocyte-C4d levels as a lupus activity marker still waits for future validation. Furthermore, potential biomarkers for monitoring lupus activity reviewed by Lateef A and Peri M are recognized as only a research tool and not yet validated for clinical use [35]. Hence, novel lupus activity biomarkers are much needed as potential parts of a combination panel with different biomarkers to predict clinical outcomes of systemic lupus erythematosus [36]. Consecutively, our research results on B-cell sialyltransferase and neuraminidase levels/ratios and sialic acid levels in lupus have never been tested before and may potentially play a role in monitoring disease activity of such a heterogeneous disease like SLE and may help to improve clinical treatment decision in SLE patients.

Since its implementation for clinical evaluation of RA disease activity, DAS28 scores have been extensively validated and widely used by rheumatologists around the world [37]. However, ESR and CRP used in the DAS28 scores have been noted in the normal range in up to 40% patients with RA [38, 39]. Hence, quite a few other biomarkers still were examined in the past two years to correlate with DAS28 sores since biomarkers could provide objective assessments of the RA disease process more than evaluation of swollen joint count, tender joint count and visual analog scale for general health. For instance, serum asymmetric dimethyl-L-arginine, soluble E-selectin, TNF-α, and IL-6 were shown to be higher in the high DAS28 score group than in the moderate and low DAS28 score group [40]. Serum CXCL 13 levels were demonstrated to differ in DAS28 remission from its DAS28 active group [41]. Both reports did not report their linear correlation and area-under-the receiver operating characteristic curve. Moreover, serum levels of progranulin, microRNA-223, and leucine-rich alpha-2 glycoprotein, and leucocyte complement receptor 1transcript have been shown to correlate with DAS28 scores, however, no one has been validated yet [42–45].

Furthermore, a new multibiomarker score has been devised to assess RA disease activity through validation with linear correlation, area-under-the receiver operating characteristic curve, and treatment response [46, 47]. In particular, treatment targeting normalization of matrix metalloproteinase 3 together with DAS28 scores down to below 2.6 has been shown to achieve better clinical effects than either one [48]. The latter report probably stresses again the importance of a combination of different biomarkers in monitoring RA disease activity. Another report on blood monocyte chemotactic protein-1 (MCP-1) and its incorporation into the DAS28 equation to make adapted DAS28-MCP-1 has demonstrated its very high correlation with DAS28 and DAS28-CRP [29]. However, it has not been validated through roentgen confirmation and functional assessment of patient daily activities. Altogether, no single test mentioned above, except for DAS28 scores, has been adopted and used worldwide now, probably due to individual test’s inherent drawbacks and/or inconvenience. Hence, novel RA activity biomarkers are still needed as parts of a combination panel to predict RA disease activity. Similarly, in turn, our research findings on B-cell sialyltransferase and neuraminidase levels/ratios and sialic acid levels (the latter did show correlation across different DAS28 catogories) in RA patients with high disease-activity DAS28 scores have never been tested before. A much larger population of RA patients is needed to show possible uses of B-cell sialyltransferase and neuraminidase levels/ratios in RA patients with categories of low, moderate, and high disease-activity DAS28 scores.

In summary, we found that the B-cell ST3Gal-1/Neu3 ratio was positively correlated with lupus disease activity. In particular, B-cell ST3Gal-1 and Neu 3 correlated oppositely with SLEDAI scores in SLE patients. In contrast, both ST3Gal-1 and Neu3 levels in blood B cells correlated positively with moderate and high disease-activity DAS28 scores in RA patients. Detection of these laboratory parameters might therefore open a new way for monitoring lupus activity, as an alternative to or in conjunction with C3c, C4, and anti-DNA levels, considering unsatisfactory correlation of these latter laboratory parameters with lupus manifestations except with nephritis. Nevertheless, these findings require confirmation in future large-scale cohort studies, in particular, in a different racial and ethnic population.

## Supporting Information

S1 Fig Representative flow data of 3 subject groups. Peripheral blood mononuclear cells (PBMCs) at 1x10^6^ cells in 100 μl phosphate-buffered saline (PBS) were obtained from (A) lupus (SLE) patients, (B), healthy controls and (C) rheumatoid arthritis (RA) patients as described in Patients and methods. PBMCs were then stained with phycoerythrin(PE)-conjugated mouse anti-human CD19 and with PE-conjugated mouse IgG1 *k* isotype control for background gating. While at the same time, PBMCs were stained with FITC-conjugated *Maackia amurensis* lectin (specifically binds α-2,3-SIA) and with FITC-mouse IgG1 *k* isotype control for background gating. After subtracting the background staining, the mean fluorescence intensity (MFI) of stained B cell-α-2,3-SIA revealed: SLE 18.5, healthy controls 50.4 and RA 76.0. These MFI mirrored the group comparison results in Fig 2B.
(TIF)

S2 Fig Different expression levels of neuraminidases in separate lupus manifestations and disparate disease activity categories. Polymorphonuclear cells (PMN) (or monocyte) Neu3 levels indicated the mean fluorescence intensity (MFI) of PMN’s (or monocyte’s) Neu3 staining results. (A) Higher monocyte Neu3 levels were found in the proteinuria subgroup (+) (n = 18) vs. the non-proteinuria subgroup (-) (n = 61) (*P*<0.001). (B) Higher PMN Neu3 levels were noted in the SLEDAI > 7 subgroup (+) (n = 16) vs. the SLEDAI ≤ 7 subgroup (-) (n = 96) (*P*<0.001). (C) Higher monocyte Neu3 levels were noted in the SLEDAI > 7 subgroup (+) (n = 16) vs. the SLEDAI ≤ 7 subgroup (-) (n = 95) (*P* <0.001).
(TIF)

S1 Table Frequencies of individual and combined medications in RA patients. (DOC)

S2 Table Frequencies of individual and combined medications in SLE patients. (DOC)
