# Supplementary figures and images for: Sialyltransferase and Neuraminidase Levels/Ratios and Sialic Acid Levels in Peripheral Blood B Cells Correlate with Measures of Disease Activity in Patients with Systemic Lupus Erythematosus and Rheumatoid Arthritis: A Pilot Study
Source: PLoS One. 2016 Mar 16;11(3):e0151669. doi: 10.1371/journal.pone.0151669 (PMC4794174; doi:10.1371/journal.pone.0151669)

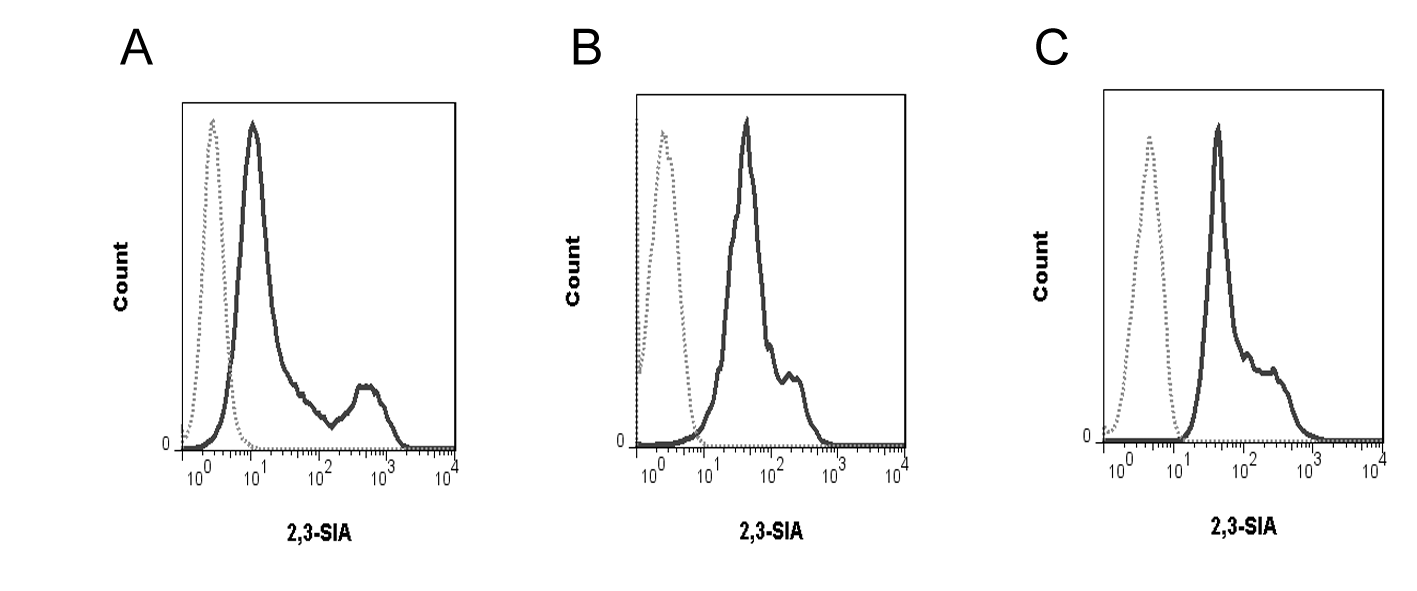

Supplement: S1 Fig — Peripheral blood mononuclear cells (PBMCs) at 1x10^6^ cells in 100 μl phosphate-buffered saline (PBS) were obtained from (A) lupus (SLE) patients, (B), healthy controls and (C) rheumatoid arthritis (RA) patients as described in Patients and methods. PBMCs were then stained with phycoerythrin(PE)-conjugated mouse anti-human CD19 and with PE-conjugated mouse IgG1 k isotype control for background gating. While at the same time, PBMCs were stained with FITC-conjugated Maackia amurensis lectin (specifically binds α-2,3-SIA) and with FITC-mouse IgG1 k isotype control for background gating. After subtracting the background staining, the mean fluorescence intensity (MFI) of stained B cell-α-2,3-SIA revealed: SLE 18.5, healthy controls 50.4 and RA 76.0. These MFI mirrored the group comparison results in Fig 2B. (TIF) [file pone.0151669.s001.tif]

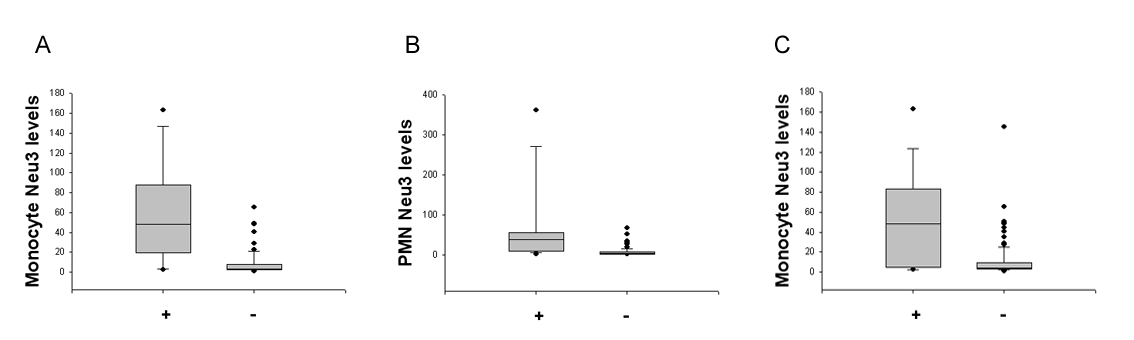

Supplement: S2 Fig — Polymorphonuclear cells (PMN) (or monocyte) Neu3 levels indicated the mean fluorescence intensity (MFI) of PMN’s (or monocyte’s) Neu3 staining results. (A) Higher monocyte Neu3 levels were found in the proteinuria subgroup (+) (n = 18) vs. the non-proteinuria subgroup (-) (n = 61) (P<0.001). (B) Higher PMN Neu3 levels were noted in the SLEDAI > 7 subgroup (+) (n = 16) vs. the SLEDAI ≤ 7 subgroup (-) (n = 96) (P<0.001). (C) Higher monocyte Neu3 levels were noted in the SLEDAI > 7 subgroup (+) (n = 16) vs. the SLEDAI ≤ 7 subgroup (-) (n = 95) (P <0.001). (TIF) [file pone.0151669.s002.tif]
