# Supplementary material for: Sialyltransferase and Neuraminidase Levels/Ratios and Sialic Acid Levels in Peripheral Blood B Cells Correlate with Measures of Disease Activity in Patients with Systemic Lupus Erythematosus and Rheumatoid Arthritis: A Pilot Study
Source: PLoS One. 2016 Mar 16;11(3):e0151669. doi: 10.1371/journal.pone.0151669 (PMC4794174; doi:10.1371/journal.pone.0151669)
Supplement: S1 Table — (DOC) [file pone.0151669.s003.doc]

**S1 Table.** **Frequencies of individual and combined medications in RA patients**

|  | Number of patients (%) | Mean dose (mg/day, except where indicated) ± S.D. (range) |
| --- | --- | --- |
| SLZ | 116 (73.9) | 1173.73 ± 352.10 (250-1500) |
| MTX | 108 (68.8) | 12.34 ± 4.39 mg/wk (5-20) |
| HCQ | 59 (51.3) | 340.16 ± 93.67 (200-500) |
| Prednisolone | 63 (40.1) | 5.78 ± 3.57 (1.25-15.00) |
| Leflunomide | 23 (14.6) | 16.20 ± 4.82 (10-20) |
| Cyclosporine | 5 (3.2) | 206.25 ± 54.77 (150-300) |
| Azathioprine | 4 (2.5) | 118.75 ± 23.66 (100-150) |
| Methylprednisolone | 3 (1.9) | 5.60 ± 2.31 (4-8) |
| Golimumab | 19 (12.1) | 50mg/mo ± 0 (50-50) |
| Adalimumab | 12 (7.6) | 78.00mg/mo ± 3.76 (70-80) |
| Etanercept | 10 (6.4) | 191.67mg/mo ± 15.81(150-200) |
| Abatacept | 3 (1.9) | 600.00mg/mo ± 144.34 (500-750) |
| SLZ + MTX | 81 (51.6) |  |
| SLZ + MTX + HCQ | 25 (15.9) |  |
| SLZ + MTX + Prednisolone | 36 (22.9) |  |
| SLZ + MTX + Leflunomide | 13 (8.3%) |  |
| SLZ + MTX + Golimumab | 12 (7.6) |  |
| SLZ + MTX + Adalimumab | 8 (5.1) |  |
| SLZ + MTX + Etanercept | 5 (3.2) |  |
| SLZ + MTX + Abatacept | 2 (1.3) |  |

RA: rheumatoid arthritis; SLZ: Sulfasalazine; MTX: Methotrexate; HCQ: Hydroxychloroquine. Data are shown as number of patients (percentage) or mean dose (mg/day, except where indicated) ± S.D. (range).
