# Supplementary material for: Sialyltransferase and Neuraminidase Levels/Ratios and Sialic Acid Levels in Peripheral Blood B Cells Correlate with Measures of Disease Activity in Patients with Systemic Lupus Erythematosus and Rheumatoid Arthritis: A Pilot Study
Source: PLoS One. 2016 Mar 16;11(3):e0151669. doi: 10.1371/journal.pone.0151669 (PMC4794174; doi:10.1371/journal.pone.0151669)
Supplement: S2 Table — (DOC) [file pone.0151669.s004.doc]

**S2 Table.** **Frequencies of individual and combined medications in SLE patients**

|  | Number of patients (%) | Mean dose (mg/day, except where indicated) ± S.D. (range) |
| --- | --- | --- |
| HCQ | 103 (89.6％) | 318.1±91.6 (100.0-400.0) |
| Pred | 88 (76.5％) | 11.7±8.4 (2.5-50.0) |
| MTX | 12 (10.4％) | 8.9±2.9/wk (5.0-15.0) |
| AZA | 25 (21.7％) | 124.1±48.6 (50.0-250.0) |
| HCQ + Pred | 53 (46.1％) |  |
| HCQ +AZA | 2 (1.7％) |  |
| HCQ + Pred + MTX | 6 (5.2％) |  |
| HCQ + Pred + AZA | 16 (13.9％) |  |
| HCQ + Pred + MTX + AZA | 5 (4.3％) |  |
| HCQ+MTX | 1 (0.9％) |  |
| AZA+Pred | 1 (0.9％) |  |

SLE: systemic lupus erythematosus; HCQ: Hydroxychloroquine; Pred: Prednisolone; MTX: Methotrexate; AZA: Azathioprine. Data are shown as number of patients (percentage) or mean dose (mg/day, except where indicated) ± S.D. (range).
